# Identification of Estrus in Sows Based on Salivary Proteomics

**DOI:** 10.3390/ani12131656

**Published:** 2022-06-28

**Authors:** Chenlei Li, Chenglei Song, Kunlong Qi, Yingke Liu, Yaqing Dou, Xiuling Li, Ruimin Qiao, Kejun Wang, Xuelei Han, Xinjian Li

**Affiliations:** College of Animal Science and Technology, Henan Agricultural University, Zhengzhou 450002, China; fubukilcl@163.com (C.L.); songchenglei99@163.com (C.S.); chyikunlong@gmail.com (K.Q.); liuyingke1998@163.com (Y.L.); dyq00713@163.com (Y.D.); xiulingli@henau.edu.cn (X.L.); qrm480@163.com (R.Q.); wangkejun.me@163.com (K.W.)

**Keywords:** Large White sow, estrus identification, salivary proteomics, protein interaction

## Abstract

**Simple Summary:**

The conception rate of sows is an important factor affecting the efficiency and income of pig farms. At present, the traditional estrus identification methods used in production, such as the external observation method and back pressure reaction method, have the disadvantages of being time-consuming and laborious, with low accuracy and high requirements for technicians. In addition, the abnormal estrus status of sows, such as continuous estrus and short estrus, will also lead to misjudgment during estrus inspection, which will affect the conception rate of sows and reduce production efficiency. Therefore, the purpose of this study was to explore a new method for estrus identification, to provide an important reference for accurate estrus identification of sows by screening salivary proteins related to estrus, and to provide a reference for the development of kits/strips for estrus identification of sows.

**Abstract:**

The estrus cycle of multiparous Large White sows was divided into three stages to solve the problems of heavy workload and low accuracy of the traditional estrus identification method in pig production. Saliva protein was extracted from the oral saliva of multiparous sows. Label-free quantitative proteomics was used to detect salivary proteome, and MaxQuant software was used for quality control. Results showed that 246 proteins were identified in the three stages, where 40 proteins were significantly different (*p* < 0.05). The total proteins identified were enriched by STEM software and the protein function was annotated by using the ClueGO plug-in in the Cytoscape software. The results were enriched to eight different trends. The annotated items were related to protein synthesis and processing and estrogen response. Gene ontology and the Kyoto Encyclopedia of Genes and Genomes enrichment analysis of differential proteins involved in the pathways and entries included oocyte meiosis, response to estradiol, and oogenesis. Further interaction analysis showed that an interaction occurred between P00355, F1SHL9, P28491, F1SDR7, F2Z558, F1RYY6, and F2Z5G3 proteins. The findings served as a basis for revealing the changes in salivary protein content in the sow estrus cycle and provided a reference for the development of an estrus identification kit/test strip in the next step.

## 1. Introduction

In the process of pig production, the accuracy of estrus identification is an important factor affecting the reproductive performance of sows. The currently used estrus identification methods, such as the external observation method, back pressure reaction method, and boar estrus test method, have the disadvantages of large workload and low accuracy. Some atypical estrus conditions, such as quiet estrus, feeble estrus and continuous estrus, are difficult to grasp for the mating timing, and have low identification accuracy and too many repeated mating times. These factors will also lead to the waste of mating resources [1]. Research on new estrus identification methods of various livestock has never stopped. The differentially expressed biomolecules in saliva [2], blood [3], urine [4], and other body fluids have become an important target for diagnosing the estrus of female livestock.

Saliva is mainly produced by three pairs of salivary glands, namely, parotid gland, sublingual gland, and mandibular gland, and a large number of small salivary gland secretions under the oral mucosa, mainly including water, some nonprotein organic substances (such as uric acid, bilirubin, creatinine, glucose, fatty acid, etc.) and some protein or polypeptide substances. It is composed of amylase, albumin, secretory immunoglobulin A, and casein rich protein. Saliva also contains some hormones (such as cortisol, testosterone, luteal hormone, estradiol, aldosterone, etc.) and nucleic acid substances (such as miRNA) [5,6]. The composition of saliva is complex. Most metabolites, hormones, antibodies and other substances in the human body can enter saliva through passive diffusion or active transport of blood. Saliva is an important and necessary body fluid in animals. It is rich in steroid hormones, DNA, high molecular sensitivity RNA, oxidative stress markers, and other components. Estrogen stimulates and maintains the development of the female reproductive tract, enables the development and maintenance of secondary sexual characteristics, stimulates follicular development, and induces estrus behavior. The obviousness of estrus signs in sows can determine their reproductive performance. In production, estrus is often identified by external observation of the sow’s behavior and physiological status. The level of estrogen in the blood is closely related to the estrus symptoms of sows. The swelling degree of the vulva, the redness of the color of the mucous membrane of the vulva and vagina, the amount and consistency of the mucus, and the acceptance of the static reflex are directly proportional to the estrogen content. In recent years, saliva has become an effective tool for detecting the physiological and pathological statuses of humans and animals in addition to plasma, serum, and urine [7]. Human saliva contains more than 2000 different proteins and more than 2000 low molecular weight polypeptide fragments, accounting for 40–50% of the secreted proteins. About 27% of the proteins in saliva and blood are the same [8]. The proteins discovered as candidate markers of tumors and cardiovascular diseases accounted for 40%. Before the last century, saliva has been used by humans for disease detection and health assessment. Khurshid et al. [9] found that the E2 content in the saliva and blood of women is directly proportional to and significantly correlated with the size and number of follicles by enzyme-linked immunosorbent assay. Therefore, the plasma free hormone level can be reflected by detecting the E2 content in saliva, which can be used in clinical practice. Hofman et al. [10] confirmed that saliva is an accurate clinical index that can be used to measure the content of free hormone and diagnose the poor function of the hypothalamic–pituitary–adrenal axis. At present, the application of saliva detection in animals is mainly used for pig disease detection, mainly including antigen and antibody detection of pig disease pathogens, such as porcine reproductive and respiratory syndrome virus, swine influenza virus, swine foot-and-mouth disease virus, porcine circovirus type 2, and classical swine fever virus [11,12,13,14,15]. In recent years, many studies have confirmed that the contents of hormones, drugs, antibodies, and viruses in saliva and blood have high similarity [14,16,17,18,19,20,21]. At present, animal saliva is mainly collected by the Pasteur pipette suction method, the natural flow method and the chewing cotton column method. When collecting animal saliva, it is necessary to ensure that the animal’s mouth is clean. Collection before feeding is optional. The saliva collected should be filtered or centrifuged to remove food crumbs and other substances. If conditions permit, an appropriate amount of protease inhibitors can be added and stored at a low temperature for reserve [22,23]. Compared with traditional blood collection, saliva collection has obvious advantages, such as simplicity, noninvasiveness, and easy storage and transportation of samples. Animals are in a natural state during saliva collection. Compared with passive blood collection, saliva collection can more truly reflect the state of animal machinery and significantly reduce the stress response of animals [24]. Saliva detection is a hot spot in noninvasive detection, which has great development potential and application prospects. At present, the estrus identification of sows by salivary protein markers has not been reported.

This study revealed the changes of salivary protein levels in sows during the estrus cycle, screened the salivary proteins related to estrus, and serves as guide for the accurate estrus identification of sows, providing a reference for the development of an estrus identification kit/test strip as the next step through the proteomic sequencing and analysis of saliva samples from different stages of the estrus cycle of Large White sows.

## 2. Materials and Methods

### 2.1. Test Animals and Sample Collection

Four Large White sows with three births were selected. The type of pig house was a fully closed pig house and a large circle feeding mode. Each circle contained 8–10 sows. The temperature in the pig house was controlled at 15–22 °C, and the humidity as maintained at 45–60%. The feeding environment as suitable, and the reproductive function was normal, healthy and free of genetic diseases. For estrus identification, we adopted the external observation method and the back pressure reaction to comprehensively identify the estrus of sows. During the test, the experimental sows were observed every morning, noon, and evening for 30 min each time. When the sows had restlessness, anorexia, pudendal congestion, swelling, mucus secretion, and climbing over other pigs, boar odorant was sprayed on the pigs’ nose, and their backs were hard-pressed. The sow standing still and bowing its back and ears was identified as estrus, and the day of estrus was recorded as day 0. The normal estrus cycle for sows is 21 days, and the anterior pituitary gland secretes follicle-stimulating hormone follicle stimulating hormone at about 18–20 days late in the estrus cycle (2 days before estrus). Luteinizing hormone peaks within 8 h of the onset of estrus (static response), estrogen peaks on estrus day, and progesterone levels are low during estrus [25]. So, we chose to collect saliva in three stages with obvious changes in reproductive hormone content before and after estrus, including PE: 3 days before estrus, E: the day of estrus, and AE: 3 days after estrus. Saliva was collected every morning before feeding (6:00 a.m.–7:30 a.m.). Using pigs’ curiosity and chewing habits, medical absorbent cotton was used to collect saliva. The absorbent cotton was made into a ball and fixed at the front end of a metal wire. The pig would chew the gauze ball actively and the saliva would enter the absorbent cotton ball through siphoning (Supplementary material Figures S2 and S4). The saliva in the absorbent cotton ball was extruded and released into the sample bag (Supplementary material Figures S1 and S3), and then the saliva in the sample bag was sucked into the centrifugal tube with a syringe, and the supernatant was collected after 4000× *g* low-temperature centrifugation at 4 °C and stored in the refrigerator at −80 °C for subsequent use of label-free quantitative proteomics technology for saliva proteome sequencing.

### 2.2. Sample Digestion and Enzymatic Hydrolysis

The saliva of four sows in the three stages was collected and transferred to three Ku ultrafiltration tubes in accordance with different stages. LABC (1 mL 25 mmol) was added and centrifuged at 4 °C 7000× *g* for 30 min three times. The liquid retained on the upper layer of the filter membrane (about 100 mL) was absorbed. Each sample was added to 100 μL SDT lysate, placed in a 100 °C metal bath for 3 min, and centrifuged, and the supernatant was collected. The sample protein lysate at −80 °C was collected and freeze-thawed, and 1 μL was quantified by the BCA method. In accordance with the quantitative results, 20 μg protein sample was taken for SDS-PAGE electrophoresis. Each sample (300 μg) was taken for formate-assisted fast pyrolysis enzymatic hydrolysis. The sample (200 μL) was mixed and shaken with UA buffer (8 mol urea, 150 mmol Tris HCl, pH 8.5), and centrifuged at room temperature for 30 min at 14,000× *g*. The filtrate was discarded, and this step was repeated three times. IAA (100 μL, 50 mmol IAA in UA) was shaken at 600 r/min for 1 min and 300 r/min at room temperature, incubated for 30 min, and centrifuged for 14,000× *g* at room temperature for 30 min. UA buffer (100 μL) was added, centrifuged for 14,000× *g* at room temperature for 30 min, and repeated three times. LABC (100 μL 25 mmol) was added, centrifuged under the same conditions, and repeated three times. Trypsin buffer (40 μL, 2 μg Trypsin in 40 μL 100 mmol/LABC) was added and placed in a constant temperature blender (300 r/min, 18 h, 37 °C) after the filtrate was discarded. The filtrate was centrifuged for 14,000× *g* at room temperature for 30 min, the filtrate was collected, and 100 mmol/L ABC was added to the filtrate at room temperature (14,000× g) for 30 min. The filtrate was centrifuged for 14,000× *g* at room temperature for 30 min. OD280 peptide was quantified.

### 2.3. Mass Spectrometry Analysis

The samples were desalted and separated by capillary high-performance liquid chromatography and analyzed by mass spectrometry with an OrbitRAP-Elite mass spectrometer after the enzymatic hydrolysis. A total of 1 μg per sample was loaded on a Thermo Scientific EASY column (two columns) by using an automatic sampler at a flow rate of 150 nL/min. The mass charge ratio of peptides and peptide fragments was collected by MS2 scan (10 fragments) after each full scan. MS2 Activation Type: CID, Isolation Window: 1 *m/z*, the mass spectrometer was running in positive ion mode, and the scanning range of the parent ion was 300–2000 *m*/*z*. Orbitrap Elite scans were conducted at 200 *m*/*z* at primary and secondary ms resolutions of 60,000 and 15,000 at *m*/*z*, respectively. The 10 strongest signals in the obtained mass spectrum were selected for further analysis.

### 2.4. Database Retrieval, Protein Identification, and Quantification

The system used for LC–MS/MS mass spectrometry was the ABSCIEX Triple TOFTM 5600 Plus mass spectrometry system, ABSCIEX analysis column, and standardized mass spectrometry data flow was used for analysis. For identified proteins, a protein with an unused score ≥ 1.3 (confidence level above 95%) and containing at least one unique peptide for each protein was defined as a trusted protein. Fold change (FC) was calculated in accordance with the label-free quantification (LFQ value) and Persus software (Version 1.4.1.3, http://www.coxdocs.org/doku.php?id=perseus:start (accessed on 12 September 2021). The *p*-value of the FC significant parameter was calculated. MaxQuant software was used to analyze the data, and the mass spectrometry data were transformed from the spectral peak to the direct 2D gel map. Each point represented a peptide segment. The relative protein of the peptide segment was quantified by comparing the strength of the corresponding peptide segments on different samples, and the peptide matching map FDR < 0.01 and peptide FDR < 0.01 were selected for screening [26].

### 2.5. Data Analysis

In this study, STEM software (http://www.sb.cs.cmu.edu/stem/ (accessed on 20 September 2021)) was used to cluster the expression data sampled in time sequence and analyze their expression pattern [27]. The function of proteins in each cluster was annotated by using the ClueGO plug-in (v. 2.5.8) in Cytoscape software (v. 3.7.2) to determine the enriched Gene Ontology (GO) terms [28] (*p* < 0.05). The identified proteins were calculated in terms of the FC and *p* value through the LFQ value and were screened in accordance with the upregulation of expression multiple ≥ 2 and *p* < 0.05. The UniProt protein database (www.uniprot.com (accessed on 10 October 2021)) was used for analysis, and three comparison groups were set: E vs. AE, E vs. PE, and AE vs. PE. The screened differential proteins were annotated with GO function and analyzed by the Kyoto Encyclopedia of Genes and Genomes (KEGG) signal pathway to screen the GO function and the KEGG signal pathway enriched by differential proteins, and the string database (http://string.embl.de/ (accessed on 23 October 2021)) in Cytoscape (http://www.cytoscape.org/ (accessed on 10 November 2021)) Software was used for the interaction network analysis of differential proteins [29].

## 3. Results

### 3.1. Identification of Salivary Proteins in the Oestrus Cycle of Large White Sows

**Figure 1:**
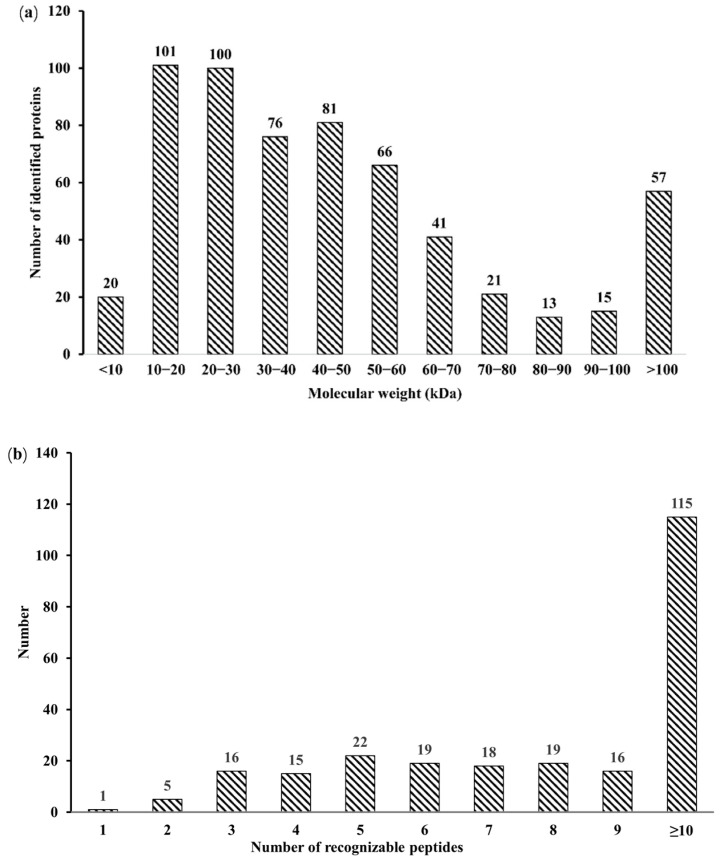
Identification and analysis of salivary proteins during the estrus cycle. (**a**) Number of peptides with different molecular weights. (**b**) Number of proteins recognizing different peptides.

**Figure 2:**
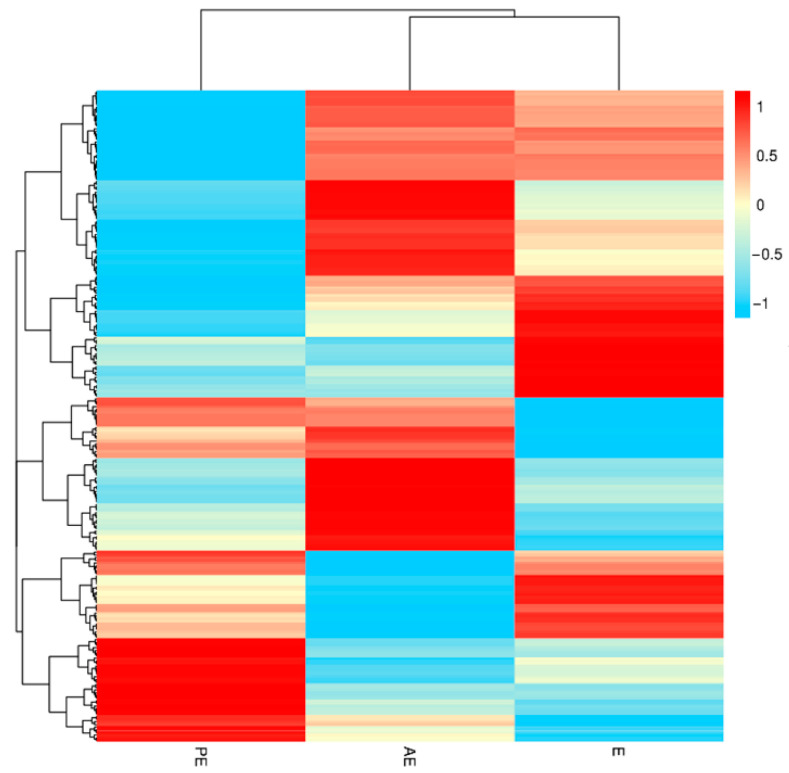
Heat map of salivary protein clustering during estrus cycle.

A total of 3405, 2179, and 2589 peptides were obtained through the analysis of salivary proteome data in the three stages of the estrus cycle, respectively, and 246 proteins were identified, including 231, 219, and 229 proteins in PE, E, and AE, respectively (Table 1). The molecular weight distribution is mainly in the range of 10–20 Ku, 20–30 Ku, 30–40 Ku, 40–50 Ku, and 50–60 Ku, accounting for 71.74% of the total protein. Among them, 97.56% of the identified proteins had three or more peptides (Figure 1a,b). The 246 identified salivary proteins were analyzed by protein cluster analysis, where the number of common proteins in PE, E, and AE was 187, and the number of common proteins in PE and E, PE and AE, E and AE were 17, 27, and 15 respectively (Figure 2).

### 3.2. Patterns of the Temporal Shifts of Salivary Proteins in the Oestrus Cycle of Large White Sows

**Figure 3:**
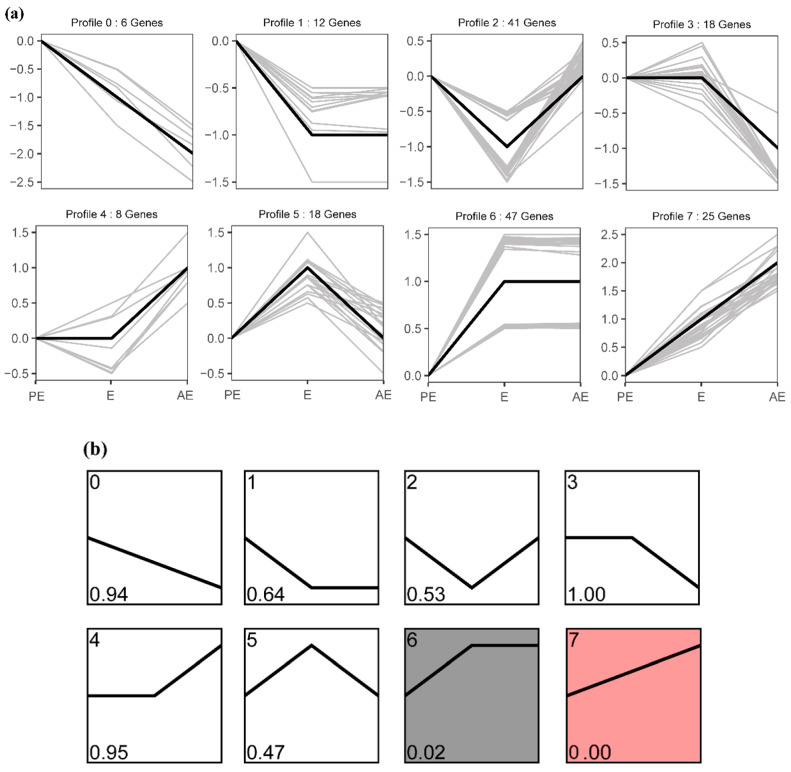
Analysis on the expression trend of salivary protein in the estrus cycle of Large White sows. (**a**) Overview of all trends. (**b**) Profiles ordered based on the *p* value significance of the number of genes assigned versus expected.

**Figure 4:**
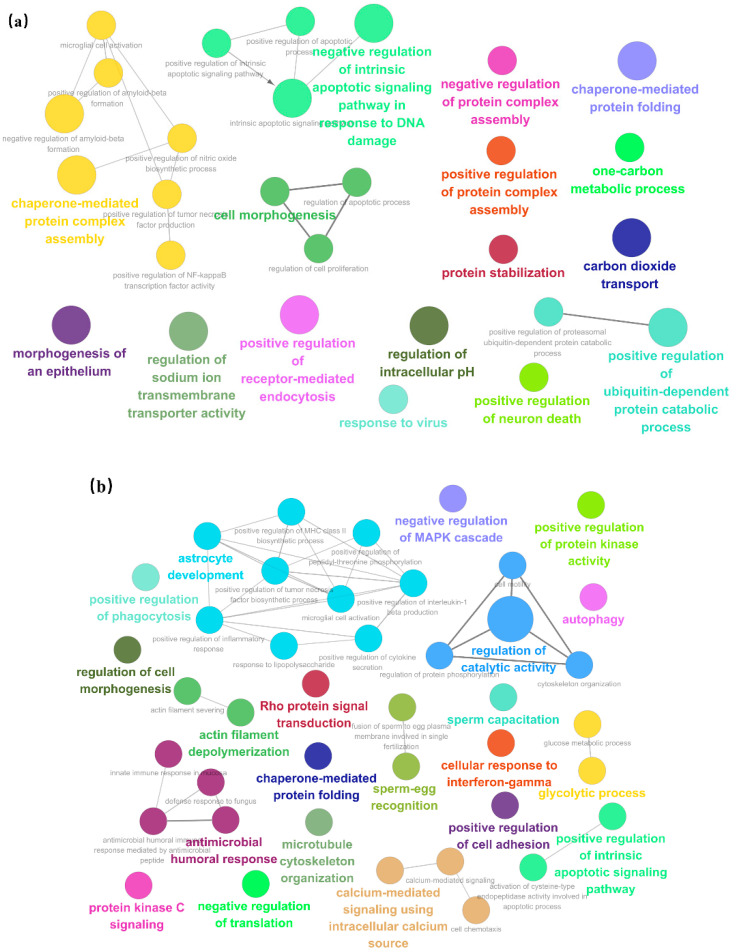

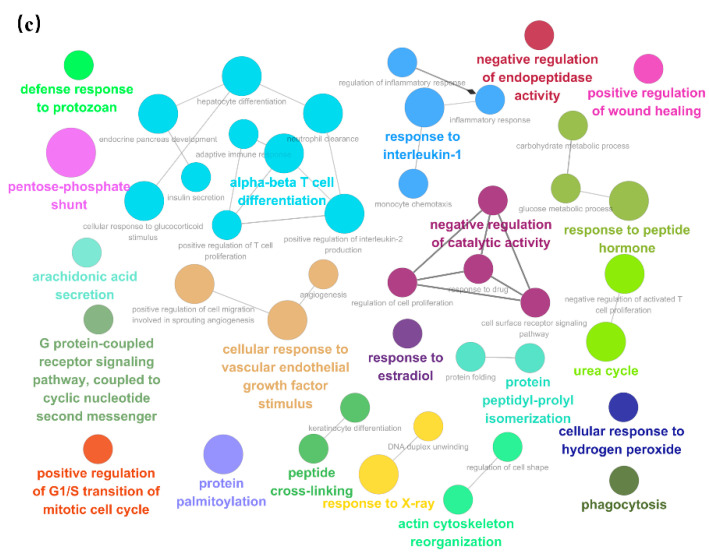
ClueGO network of GO terms. (**a–c**) represent the GO annotation results from trends 5–7, respectively. Each node represents a term, and different node colors represent various categories to which they belong.

The expression data of the identified proteins were clustered in chronological order, their expression patterns were analyzed, and eight different clusters were determined. The whole protein expression trend was divided into eight groups, which were coded from 0 to 7. Trends 6 and 7 were the significant enrichment trends (*p* < 0.05, Figure 3a,b). The protein functions in the three trends of E upregulation were annotated by using the ClueGO plug-in (v. 2.5.8) in the Cytoscape software (v. 3.7.2) to determine the enriched GO terms (*p* < 0.05, Figure 4a–c).

The proteins in trend 5 (18 proteins) were upregulated in E and downregulated in AE. Proteins in this trend are mainly involved in protein processing, synthesis, and catabolism, and some proteins are involved in cell morphogenesis, such as chaperone-mediated protein complex assembly, negative regulation of protein complex assembly, positive regulation of protein complex assembly, protein stabilization, chaperone-mediated protein folding, positive regulation of ubiquitin-dependent protein catabolic process, and cell morphogenesis (Figure 4a). The results show that a large number of proteins will be processed and synthesized in phase E sows, metabolism will be accelerated, and some proteins will participate in cell proliferation.

The proteins in trend 6 (47 proteins) remained stable after the E upregulation until AE. Proteins in this trend are mainly involved in cell signal transduction and sperm egg recognition, such as calcium-mediated signaling using an intracellular calcium source, protein kinase C signaling, positive regulation of protein kinase activity, positive regulation of intrinsic apoptotic signaling pathway, rho protein signal transduction, sperm–egg recognition, and sperm capacitation (Figure 4b). The results show that the main function of most of the proteins of trend 6 is cell signal transduction, and the subsequent sperm–egg recognition process is inseparable from the signal transmission between cells. This result also shows that the proteins of trend 6 remain unchanged after the E upregulation until the result of AE.

The proteins in trend 7 (25 proteins) are continuously upregulated in E and AE, which are mainly involved in cell proliferation and response to hormones, such as the regulation of cell proliferation, positive regulation of G1/S transition of mitotic cell cycle, alpha-beta T cell differentiation, actin cytoskeleton reorganization, positive regulation of wound healing, response to estradiol, and response to peptide hormone (Figure 4c). This result shows that hormones play a role frequently in E and AE. Growth hormone and sex hormone can promote cell proliferation and differentiation, control body growth and development and reproductive function, and affect its aging process, such as estrogen.

### 3.3. Screening and Analysis of Salivary Differential Proteins in the Oestrus Cycle of Large White Sows

**Figure 5:**
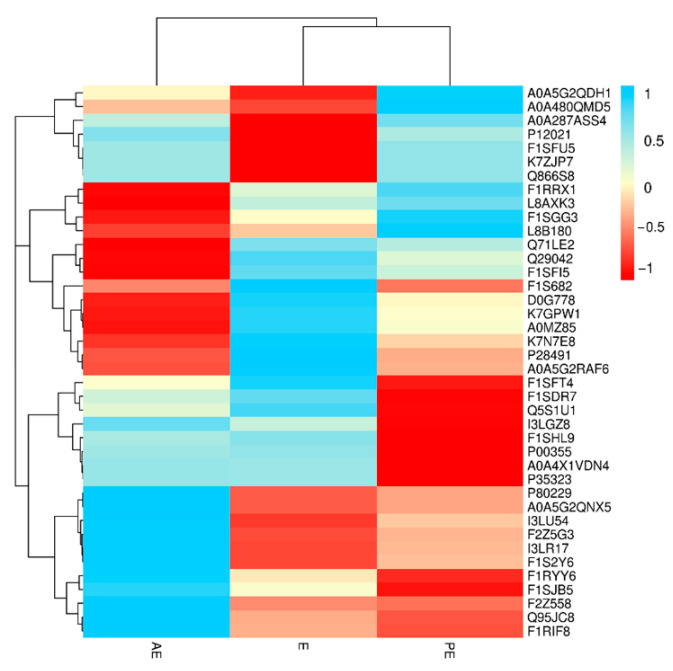
Heat map of salivary differential proteins in estrus cycle.

The difference significance analysis was conducted on the 40 differential proteins selected. As shown in Figure 5, in the E vs. PE group, 10 proteins had significant differences and were significantly upregulated (*p* < 0.05). In the E vs. AE groups, 24 significantly different proteins, including 11 upregulated and 13 downregulated proteins (*p* < 0.05) were found. In the PE vs. AE group, 11 proteins were significantly upregulated (*p* < 0.05).

### 3.4. Enrichment of Salivary Fifferential Proteins in the Estrus Cycle of Large White Sows Based on GO and KEGG Analysis

**Figure 6:**
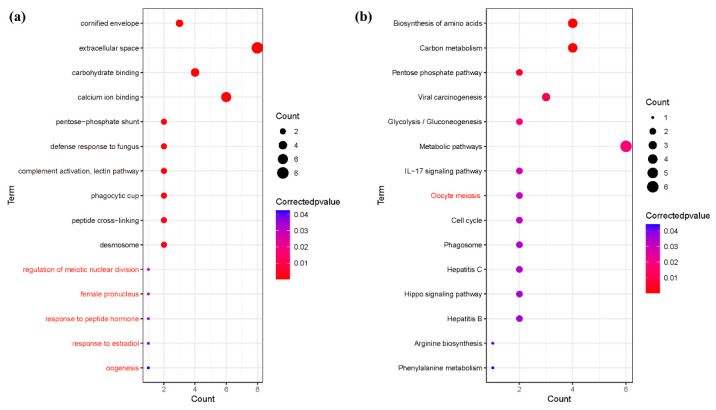
Functional enrichment analysis of different proteins in the saliva of Large White sows. (**a**) GO enrichment analysis of salivary differential proteins during the estrus cycle. (**b**) Enrichment analysis of salivary differential protein KEGG pathway during the estrus cycle.

The results of the GO and KEGG enrichment analysis of 40 differentially significant proteins showed that the GO items involved in the significant enrichment were mainly related to biological processes, where the items related to reproduction included the regulation of meiotic nuclear division, female pronucleus, response to peptide hormone, response to estradiol, and oogenesis. (*p* < 0.05), and KEGG pathway mainly includes oocyte meiosis (*p* < 0.05). The results are shown in Figure 6 (Supplementary material Tables S1 and S2).

### 3.5. Protein Interaction Network Analysis

**Figure 7:**
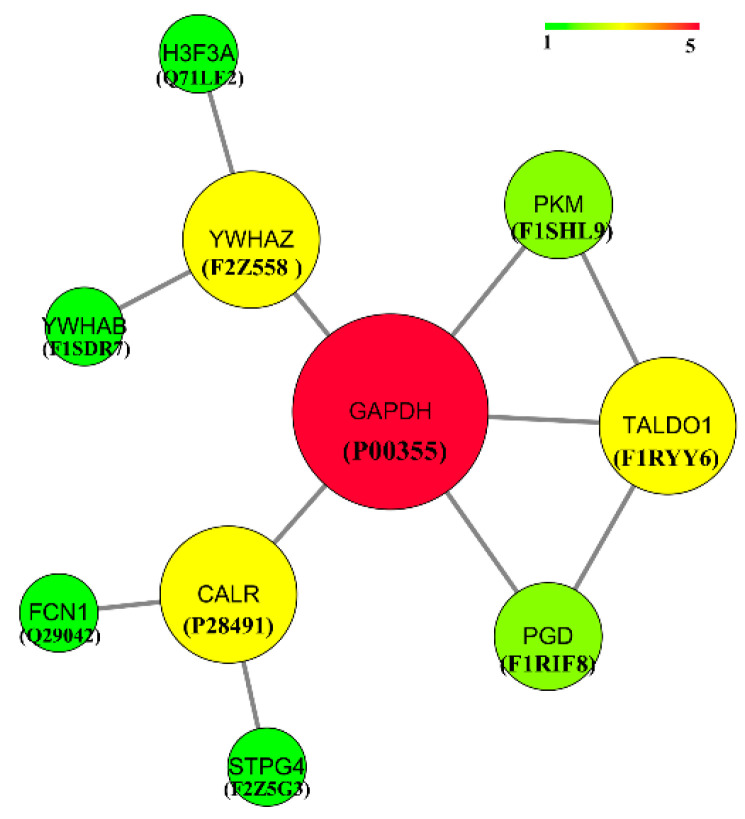
Analysis of salivary differential protein network interaction in estrus cycle.

Cytoscape software was used to analyze the protein interaction of the enriched differential and specific proteins to further reveal their functions. The results are shown in Figure 7. An interaction relationship was found between P00355 and F2Z558, P28491, F1RIF8, F1RYY6, F1SHL9, F2Z558, Q71LE2, F1SDR7, P00355, P28491, Q29042, F2Z5G3, P00355, F1RYY6, F1SHL9, F1RIF8, P00355, F1SHL9, and P00355, F1RYY6, F1RIF8 and P00355, F1RYY6. Among them, P00355 interacted with other proteins most strongly.

## 4. Discussion

The estrus cycle of sows is affected by external factors, such as temperature, light, and nutrient level, and is closely related to internal factors, such as the nervous system and endocrine system. Estrogen, progesterone, luteinizing hormone, and follicle-stimulating hormone are involved in estrus. Hormones usually work in the body by binding to receptor proteins. In different stages of the estrus cycle, the hormonal regulation of protein is different, and proteins in organisms do not exist independently. Therefore, the hormone regulation network of interactions between proteins and the related research was investigated through the interaction between different protein functions together to reveal the estrus cycle regulation mechanism. In this study, 40 significantly different proteins were screened by sequencing and analysis of salivary protein groups of sows’ estrus cycles. Ten potential salivary hormone proteins, namely, GAPDH, YWHAZ, CALR, TALDO1, PKM, PGD, H3F3A, YWHAB, FCN1, and STPG4, were screened through functional annotation and analysis. The expression patterns of P00335, P28491, F1SHL9, and F1SDR7 were the same, which were significantly upregulated in the E phase and downregulated in the AE phase. The expression patterns of F1RIF8, F1RYY6, and F2Z558 were the same and continuously upregulated in PE, E, and AE. Q29042 and Q71LE2 were significantly upregulated in the E phase and not expressed in the AE phase. F2Z5G3 was not expressed in the E phase, but expressed in the PE and AE phases.

Glyceraldehyde-3-phosphate dehydrogenase (P00355) encoded by GAPDH has glyceraldehyde-3-phosphate dehydrogenase and nitrosylase activities, thereby playing a role in glycolysis and nuclear functions. QTLs related to reproduction also existed in GAPDH. A single physiological dose of estradiol upregulates estrogen receptor α (ERα), progesterone receptor and glyceraldehyde 3-phosphate dehydrogenase (GAPDH), and lng et al. [30] speculated that these genes may be upregulated by a preovulatory surge of estrogen in sheep on the night of the fifteenth estrus cycle. Ramirez et al. [31] studied estradiol targeting several physiologically relevant membrane proteins in the central nervous system. A dose of 10 nM 17 beta-estradiol stimulated the catalysis of GAPDH, whereas progesterone at 100 nM inhibited it. Joe et al. [32] indicated that GAPDH is a target site for 17beta-estradiol and progesterone and suggested the possible roles in the regulation of cellular metabolism and synaptic remodeling in which GAPDH has been reported to be involved. These studies suggest that GAPDH affects the estrogen expression by binding the endoplasmic reticulum (ER). CALReticulin (P28491) is encoded by CALR, which is a calcium-binding chaperone that promotes folding, oligomeric assembly, and quality control in the ER via the CALReticulin/calnexin cycle. It is involved in maternal gene expression regulation and may participate in oocyte maturation via the regulation of calcium homeostasis [33]. A reproduction-related QTL was found on this CALR. Eduardo et al. [34] have shown that CRM-1 and CALR were upregulated in mammary tumors relative to normal mammary tissue. The mRNA and protein levels of CRM-1 and CALR were higher in breast cancer cells lacking ERα compared with those that express Erα. Multifunctional fusion protein (F1SHL9) is encoded by PKM. Pyruvate kinase M (PKM) is a critical regulator of this metabolic reprogramming. In a study by Salama et al. [35], E2 enhanced the PKM splicing into the PKM2 isoform by upregulating the c-Myc-hnRNP axis. PKM2 physically interacted with the ERα and functioned as an ERα coactivator. Tyrosine 3-monooxygenase (F1SDR7) is encoded by YWHAB. Zhu et al. [36] pointed out that miRNA has-miR-542-5p is associated with tamoxifen resistance. Has-miR-542-5p may be acting through a mechanism involving the target genes YWHAB. Transaldolase (F1RYY6) is encoded by TALDO1. Transaldolase is important for the balance of metabolites in the pentose-phosphate pathway. TALDO1 was identified as a key gene co-expressed with SLC1A5. Tyrosine 3-monooxygenase (F2Z558) is encoded by YWHAZ and is an enzyme responsible for catalyzing the conversion of amino acid L-tyrosine to dihydroxyphenylalanine. Frasor et al. [37] pointed out that high expression of two of the tamoxifen-stimulated genes, YWHAZ/14-3-3z and LOC441453, is found to correlate significantly with disease recurrence following tamoxifen treatment. As a protein molecule, estrogen receptor mostly exists in the cells of target organs, such as ovary and uterus. Estrogen binds specifically to estrogen receptors, thereby making estrogen exert its biological effects. Protein interaction analysis showed that YWHAZ, PKM, TALDO1, and CALR all interacted with GAPDH, and YWHAB interacted with YWHAZ. The protein quantification results showed that GAPDH, PKM, CALR, and YWHAB expression patterns were all significantly upregulated in E and downregulated in AE. YWHAZ and TALDO1 were continuously upregulated in E and AE. Therefore, it is speculated that these proteins interact with GAPDH and bind Erα, regulating the concentration of estrogen.

Calmodulin (F2Z5G3) is encoded by STPG4, which is associated with calcium-mediated signaling. Lu et al. [38] indicated that F2Z5G3 is a signaling molecule of the cAMP signaling pathway and calcium signaling pathway, and its downregulation inhibits the secretion of P4 (progesterone) while upregulating the synthesis of estradiol, a hormone secreted by the ovary, placenta, and adrenal glands at a specific physiological status. The protein quantification results indicated that F2Z5G3 is downregulated at E, which is consistent with the study of Lu et al. Therefore, F2Z5G3 may regulate the expression levels of estradiol and progesterone through the cAMP signaling pathway and affect estrus performance.

The estrus cycle of sows is about 21 days, and the estrus period lasts for 2–3 days. During the estrus period, the ovaries secrete estrogen. An important role of estrogen is to cause the estrus performance of sows. The content of estrogen gradually increases from PE to E and reaches the peak in E; most of the ovulation of sows occurs in the last 1/3 of the estrus period. The hormone playing a major role in this stage is luteinizing hormone, which is mainly used to promote the follicles to excrete eggs; luteinizing hormone peak appeared after the beginning of estrus. Ovulation occurs in sows from E to AE, the ovulation site forms corpus luteum, and the corpus luteum secretes progesterone. Therefore, the content of progesterone is low during estrus and high during diestrus. The content of estrogen and luteinizing hormone decreased gradually during this process. The expression patterns of GAPDH, PKM, CALR and YWHAB were the same, which were significantly upregulated in E and downregulated in AE. The expression patterns were also consistent with the changes of estrogen content during estrus. Therefore, it is speculated that during PE to E, the increased expression of GAPDH, CALR and YWHAB, combined with more Erα, leads to the increase of estrogen content, while E2 can enhance the splice between PKM and PKM2 by upregulating the c-Myc-hnRNP axis. PKM2 can also act as a coactivator of ERα to further increase estrogen content. From E to AE, the content of estrogen began to decrease, and the content of the corresponding four proteins also decreased correspondingly. During this period, the site of ovulation formed corpus luteum and began to secrete progesterone. It is worth mentioning that STPG4 is significantly downregulated in E and upregulated in AE, and its expression pattern is just the opposite. Downregulation of STPG4 inhibits the secretion of P4 (progesterone) and upregulates the synthesis of estradiol. Therefore, it is speculated that the upregulation of this protein from E to AE may inhibit the synthesis of estradiol and promote the secretion of progesterone through the cAMP signaling pathway and the calcium signaling pathway.

The next step is to prepare antigenic antibodies from the screened candidate proteins, an ELISA kit builds test method, test paper bar detection, and analysis of the experiment to verify the screening of this protein for the development of an estrus identification kit and to provide a solid foundation for popularization and application in production.

## 5. Conclusions

In this study, label-free quantitative proteomics was used to sequence and analyze the saliva proteome of parturient Large White sows during the estrus cycle. Seven candidate proteins in estrus were determined through differential protein screening and bioinformatics analysis. Among them, P00355, F1SHL9, P28491, F1SDR7, F2Z558, and F1RYY6 are closely related to the content of estrogen, and F2Z5G3 can regulate the amount of estrogen and progesterone through the cAMP signal pathway. The results of this study serve as reference for the development of estrus identification kits/strips in the future.

## Figures and Tables

**Table 1:** Statistics and analysis of salivary protein in different stages of estrus cycle.

Sample	MS/MS	Peptide Number	Protein Number
PE	4003	3405	231
E	2200	2179	219
AE	2813	2589	229

## Data Availability

All data generated or used during this study are available upon request from the corresponding author.

## Supplementary Materials

The following supporting information can be downloaded at: https://www.mdpi.com/article/10.3390/ani12131656/s1, Table S1: Go enrichment analysis of salivary differential protein during estrus cycle; Table S2: Enrichment analysis of salivary differential protein KEGG pathway during estrus cycle; Figure S1: Saliva is collected with less impurities; Figure S2: Sampling objects; Figure S3: The amount of saliva collected per sow; Figure S4: Sows chew absorbent cotton.
