# Supplementary material for: Identification of Estrus in Sows Based on Salivary Proteomics
Source: Animals (Basel). 2022 Jun 28;12(13):1656. doi: 10.3390/ani12131656 (PMC9264986; doi:10.3390/ani12131656)
Supplement: Supplementary file 1 [file animals-12-01656-s001.zip › animals-1742583-supplementary.pdf]

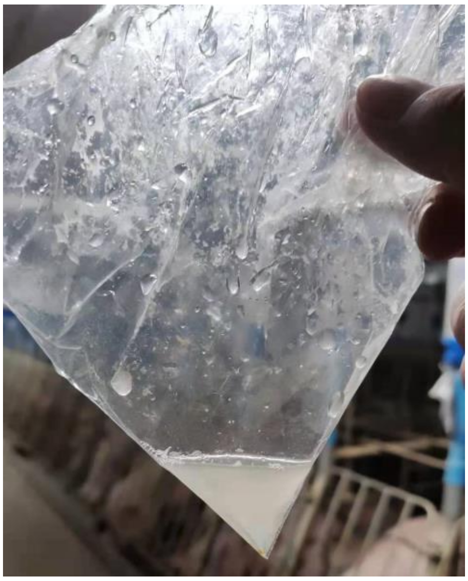

Figure S1. Saliva is collected with less impurities

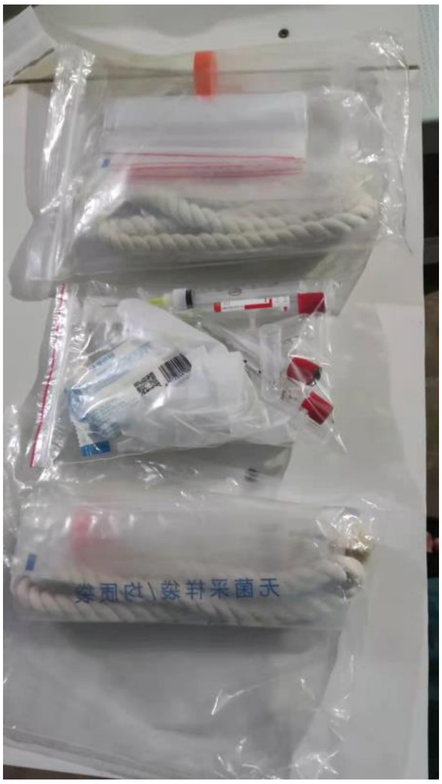

Figure S2. Sampling objects

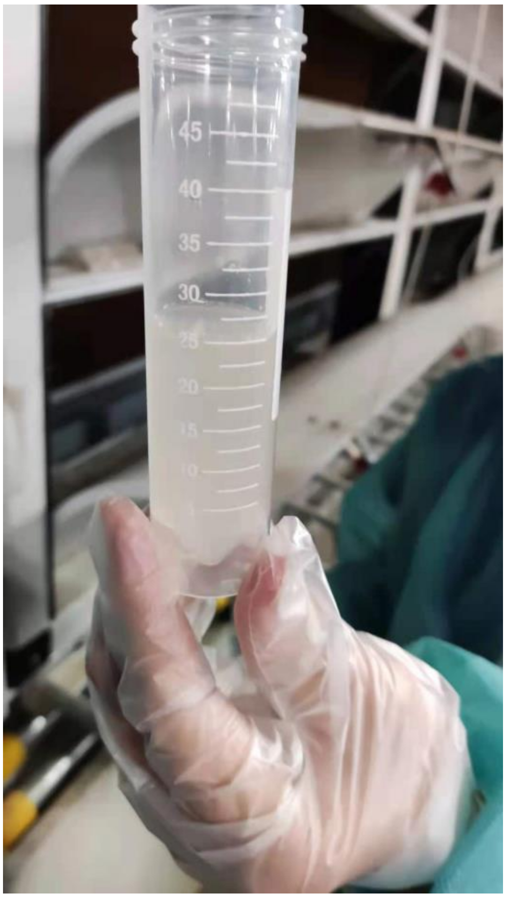


Figure S3. The amount of saliva collected per sow

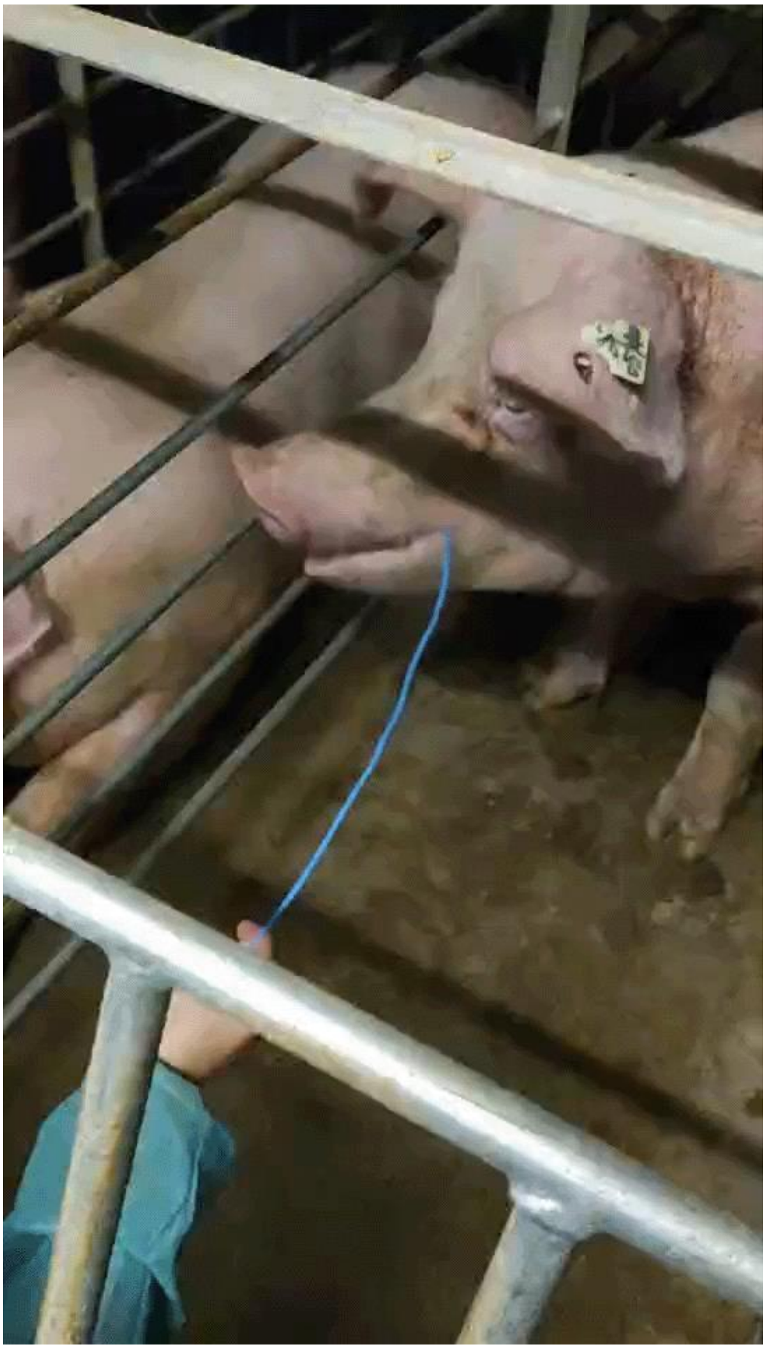

Figure S4. Sows chew absorbent cotton
